# Entry receptors — the gateway to alphavirus infection

**DOI:** 10.1172/JCI165307

**Published:** 2023-01-17

**Authors:** Ofer Zimmerman, Autumn C. Holmes, Natasha M. Kafai, Lucas J. Adams, Michael S. Diamond

**Affiliations:** 1 Department of Medicine,; 2 Department of Pathology and Immunology,; 3 Department of Molecular Microbiology, and; 4 The Andrew M. and Jane M. Bursky Center for Human Immunology and Immunotherapy Programs, Washington University School of Medicine, St. Louis, Missouri, USA.

## Abstract

Alphaviruses are enveloped, insect-transmitted, positive-sense RNA viruses that infect humans and other animals and cause a range of clinical manifestations, including arthritis, musculoskeletal disease, meningitis, encephalitis, and death. Over the past four years, aided by CRISPR/Cas9–based genetic screening approaches, intensive research efforts have focused on identifying entry receptors for alphaviruses to better understand the basis for cellular and species tropism. Herein, we review approaches to alphavirus receptor identification and how these were used for discovery. The identification of new receptors advances our understanding of viral pathogenesis, tropism, and evolution and is expected to contribute to the development of novel strategies for prevention and treatment of alphavirus infection.

## Introduction

**Figure 1:**
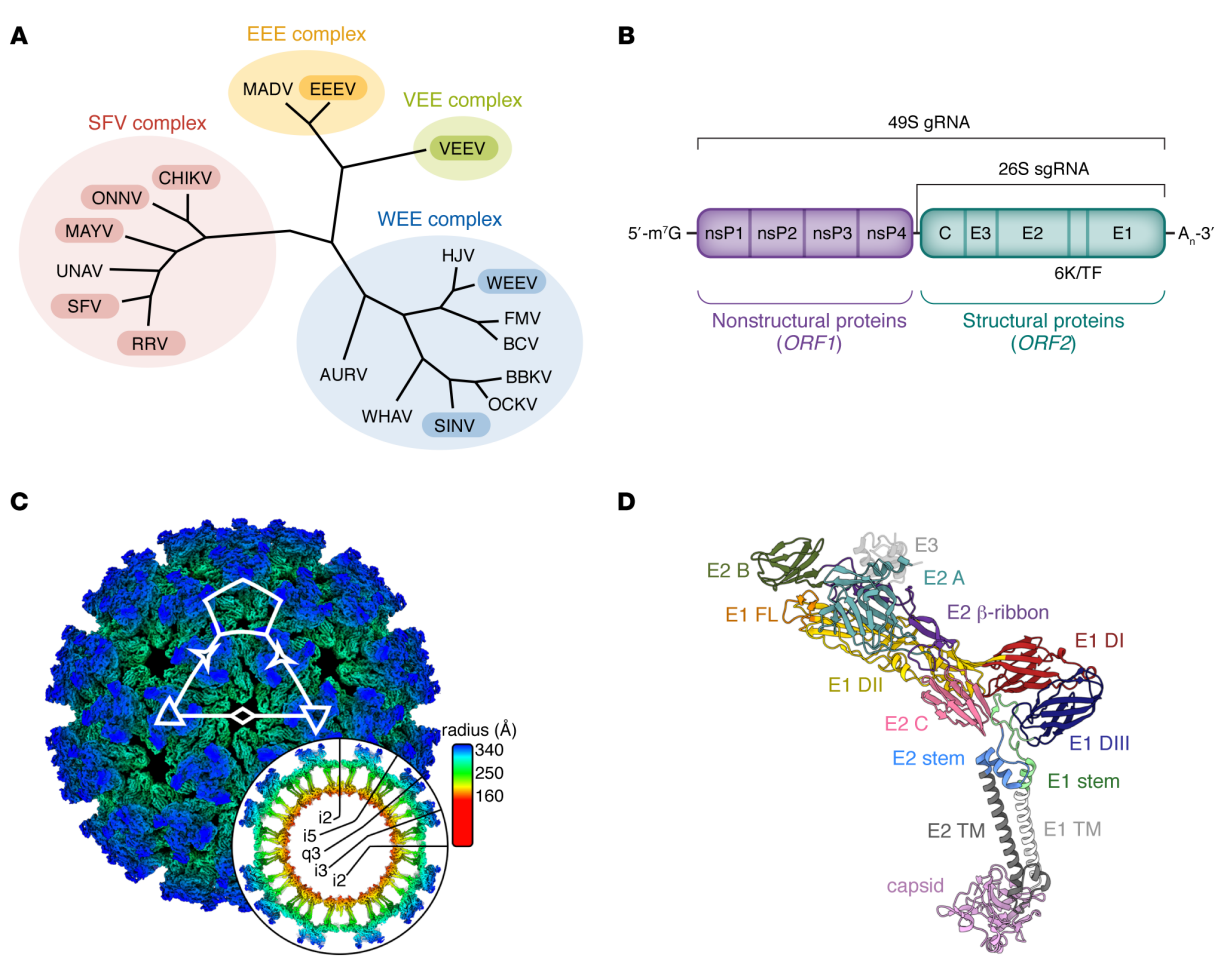
Alphavirus phylogeny, genome composition, and virion structure. (**A**) Phylogenetic tree constructed from pairwise distances between alphavirus structural protein (E1 and E2) sequences, visualized in R using the ggtree package (179). Viruses include chikungunya (CHIKV, NCBI GenBank: QKY67868.1), Mayaro (MAYV, QED21311.1), Una (UNAV, YP_009665989.1), O’nyong’nyong (ONNV, AAC97205.1), Semliki Forest (SFV, NP_463458.1), Ross River (RRV, AAA47404.1), Eastern equine encephalitis (EEEV, ANB41743.1), Madariaga (MADV, AXV43855.1), Venezuelan equine encephalitis (VEEV, AGE98294.2), Sindbis (SINV, AAM10630.1), Aura (AURV, NP_632024.1), Ockelbo (OCKV, P27285.1), Western equine encephalitis (WEEV, QEX51909.1), Buggy Creek (BCV, AEJ36227.1), Babanki (BBKV, AVN98166.1), Fort Morgan (FMV, YP_003324588.1), Highlands J (HJV, YP_002802300.1), and Whataroa (WHAV, AEJ36239.1) viruses. Viruses with known receptors are in shaded bubbles. (**B**) The alphavirus genome consists of two open reading frames, a 49S genomic RNA encoding both nonstructural and structural proteins, and 26S subgenomic RNA encoding only the structural proteins. (**C**) Cryo–electron microscopy reconstruction of VEEV virus-like particle (EMD-24117) (167) colored radially, with an equatorial cross section shown as a round inset. Axes of symmetry are designated by a pentagon (5-fold; i5), triangles (3-fold; i3), three-pointed stars (quasi-3-fold; q3), and a diamond (2-fold; i2), with axial orientations displayed in the inset. (**D**) Model of VEEV structural proteins (Protein Data Bank 7FFE), including E3, which is cleaved during viral maturation, colored by domain as indicated. Cryo–electron microscopy map and model visualized using ChimeraX (180). FL, fusion loop; TM, transmembrane. Panels **C** and **D** use structural data from Basore et al. (165) and Ma et al. (168).

Alphaviruses comprise a group of globally important enveloped, positive-sense RNA insect-transmitted viruses in the Togaviridae family (1) (Figure 1A). Two clinical syndromes occur in humans with alphavirus infection: acute encephalitis and neurological disease are caused by Venezuelan equine encephalitis (VEEV), Eastern equine encephalitis (EEEV), and Western equine encephalitis (WEEV) viruses; and acute and chronic musculoskeletal disease and arthritis are caused by chikungunya (CHIKV), Ross River (RRV), Barmah Forest (BFV), O’nyong’nyong (ONNV), Mayaro (MAYV), and Sindbis (SINV) viruses (2). Alphaviruses that infect humans can have different enzootic reservoirs, including nonhuman primates, macropods, rodents, and birds (1).

Over the last several thousand years, New World alphaviruses (e.g., VEEV and EEEV) evolved and separated from Old World alphaviruses (e.g., SINV and Semliki Forest virus [SFV]) (3) (Figure 1A). The Western equine encephalomyelitis (WEE) complex is an antigenically related group (4) that includes four New World viruses (Aura, Fort Morgan, Highlands J, and WEEV) and several Old World viruses (e.g., SINV and Whataroa) (1). WEEV is a descendant of a recombination event between a SINV-like virus and EEEV that is believed to have occurred in South America (5, 6). The three human encephalitic alphaviruses (VEEV, EEEV, and WEEV) are present only in the New World (7). The first attributed epidemic of EEEV occurred in horses in Massachusetts in 1831 (8), although EEEV was not isolated until 1933 (9). In the United States, 285 laboratory-confirmed cases of EEEV were identified in humans between 1964 and 2013 (10, 11). Between 2003 and 2018, an average of eight EEEV disease cases were reported annually in the United States (12, 13). However, in 2019, 38 cases of EEEV disease were documented in seven different states (13). The case fatality rate in symptomatic patients with EEEV infection is roughly 33%, the highest among arboviruses in the United States (14), with the majority of survivors suffering long-term neurological damage (14). VEEV has a lower case fatality rate compared with EEEV, approximately 1% (7). VEEV was isolated in 1936 from equines (15) and first documented in humans in the 1960s in Venezuela. In 1995, an epidemic with 75,000 patients occurred in Columbia with 300 deaths and 3,000 patients with neurological complications (16). Both VEEV and EEEV were weaponized as aerosol agents by the United States and the Soviet Union during the Cold War (17–19). WEEV has a case fatality rate of 3% to 7% (7). It was first isolated in 1930 in the San Joaquin Valley in California (20). Between 1964 and 2009, 639 cases of WEEV were documented in the United States.

Although CHIKV was first isolated in Tanzania in 1952, there are historical reports of epidemics of fever, arthralgias/arthritis, and rash in India dating back to 1824 (21). Indeed, epidemics of a CHIKV-like illness also were reported in the 19th century in Africa, Southeast Asia, and possibly the Americas (22). In 2003, CHIKV reemerged in islands of the Indian Ocean, including Madagascar, Comoros, Mayotte, the Seychelles, Mauritius, and La Reunion Island. Outbreaks of an Asian lineage of CHIKV in Oceania occurred in 2011 in New Caledonia. In 2012, an East/Central/South African strain of CHIKV infected more than 1,500 individuals in Papua New Guinea (23). In 2013, CHIKV spread to the Western Hemisphere, and by the end of 2015, it had infected more than 1.8 million people in North, Central, and South America (24, 25). Epidemiological estimates suggest that millions experienced acute infection and arthritis, and at least approximately 400,000 people in the Western Hemisphere suffered from chronic CHIKV arthritis (26).

The clinical phenotype of related arthritogenic alphaviruses is similar to that of CHIKV. RRV is endemic to Australia and the South Pacific Islands (27–29). The arthralgia caused by RRV infection is milder than that in CHIKV infection, and high-grade fever is less common (27). ONNV was isolated in eastern sub-Saharan Africa in the 1950s, circulates currently in sub-Saharan Africa, and has caused several major outbreaks in both East and West Africa (30). In addition to fever, joint pain, and rash, ONNV is associated with cervical lymphadenopathy (30). MAYV was isolated in South America also in the 1950s (31). The arthralgia associated with MAYV virus can last for several weeks and be incapacitating in nature, much like with CHIKV infection (31). SINV causes periodic outbreaks in Africa, northern Europe (Finland and Sweden), Asia, and Australia and has three antigenic subtypes: Babanki (Africa), Ockelbo (Sweden), and Kyzylagach (Azerbaijan and China). Infection with SINV and related viruses causes syndromes of fever, rash, and mild to moderate arthralgia in humans.

In this Review, we highlight advances in our understanding of entry receptor interactions of clinically important alphaviruses. Recent functional genomic screens with SINV, CHIKV, VEEV, and EEEV have identified new receptors, which have facilitated novel approaches to combat emerging alphavirus infections. We focus this Review on alphaviruses that are relevant to humans and other mammalian hosts. We note that there are other alphaviruses not covered in this Review, including ones that target fish that may have a large economic impact (32, 33) and insect-specific alphaviruses (e.g., Eilat and Yada Yada viruses) (34, 35).

## Alphavirus infection cycle

The approximately 11-kilobase alphavirus RNA genome encodes four nonstructural proteins (nsP1, nsP2, nsP3, and nsP4), which mediate viral translation, replication, and host immune evasion (36), and six structural proteins (capsid, E3, E2, 6K, transframe [TF], and E1). E2 and E1 are transmembrane proteins that interact to form a heterodimer (Figure 1B). The 70 nm mature alphavirus virion displays 80 trimeric E2-E1 heterodimer spikes arranged in T=4 icosahedral symmetry (37, 38) (Figure 1C). Initially, it was postulated that alphavirus E2 protein facilitated receptor engagement (39), and E1 mediated membrane fusion after viral entry (39, 40). However, more recent analysis suggests that both E2 and E1 proteins contribute to receptor engagement. E1 has three ectodomains, DI, DII, and DIII (41) (Figure 1D). E1 also contains a stem region that connects DIII to the transmembrane domain of the protein (41–43), and the hydrophobic fusion loop is located at the tip of DII. DIII adopts an immunoglobulin-like fold and is connected to DI through a linker region (44). E2 comprises three ectodomains, A, B, and C (45, 46) (Figure 1D). A subdomain D within E2 has been identified in the VEEV crystal structure (37) and observed in SINV (46), and contains residues that facilitate budding of SFV (47). Domain B is positioned furthest from the lipid bilayer, domain C is membrane proximal, and domain A is located between domains B and C (45). E2 also contains a β-ribbon motif that connects domains A and B (43, 45).

**Figure 2:**
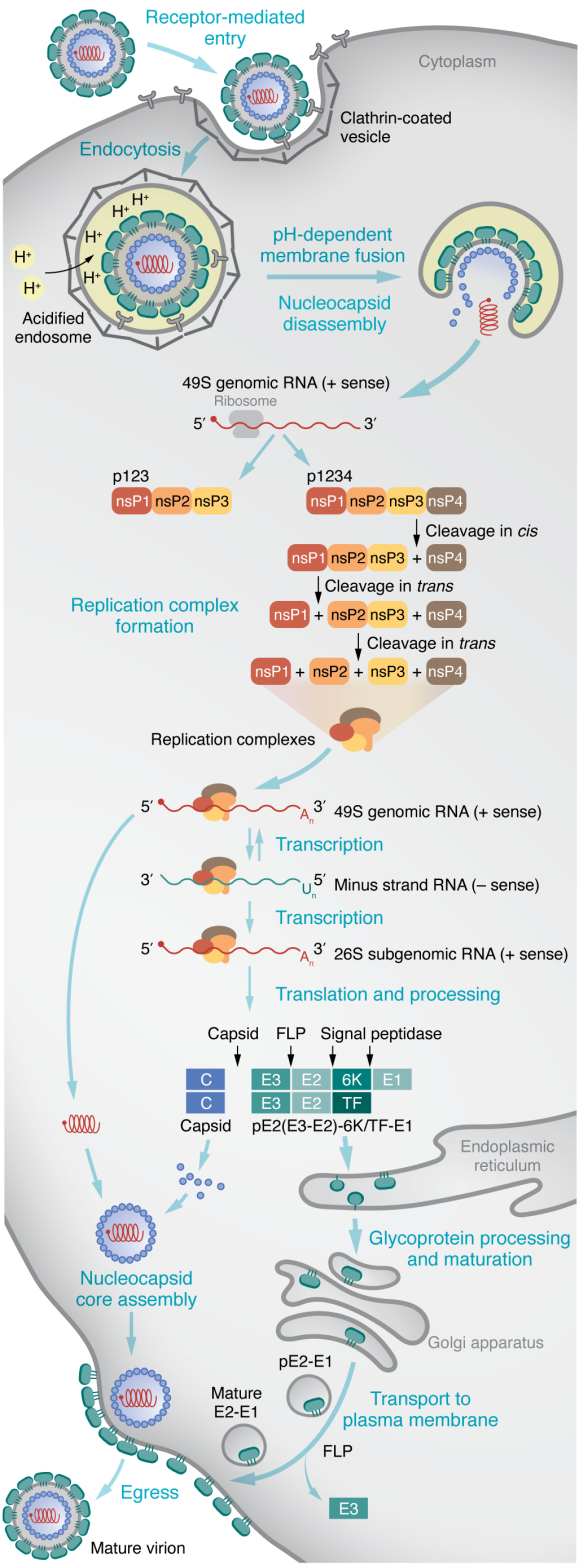
Alphavirus infection cycle. Alphaviruses can engage attachment factors (e.g., HS) and specific receptors (e.g., MXRA8, LDLRAD3, VLDLR, and ApoER2) at the cell surface to mediate binding and entry. Virions enter cells principally through endocytosis of clathrin-coated vesicles. The acidic environment of the transiting endosome triggers conformational changes in the envelope proteins, allowing for fusion with endosomal membranes, penetration into the cytoplasm where nucleocapsid disassembly occurs, and translation of the incoming positive-strand genomic RNA. At early stages of infection, genomic RNA is translated to yield P123 and P1234 polyproteins (52). P1234 is cleaved in *cis* by the nsP2 protease to generate the viral proteins necessary for transcription and replication (53, 54). The early replicase, P123 and nsP4, is processed into a short-lived nsP1, P23, and nsP4 complex and, further, into a late replicase consisting of nsP1, nsP2, nsP3, and nsP4. The early replicase synthesizes negative-strand RNA, which is used for production of genomic and subgenomic RNAs (26S) (55–58). The subgenomic mRNA drives expression of structural polyproteins C-pE2-6K/TF-E1 (56, 57). The E2 envelope glycoprotein is translated and covalently linked to E3 to form the polyprotein pE2, which associates with E1. The transframe (TF) protein is produced by ribosomal frameshift during translation of the 6K gene. The viral capsid (C) protein is released by its autoprotease activity and associates with newly synthesized genomic RNA to form the nucleocapsid. The remaining structural polyprotein is processed and matured in the endoplasmic reticulum, where host signal peptidases cleave pE2, 6K/TF, and E1 (63, 68, 70–72). Furin-like proteases (FLP) in the Golgi network cleave pE2 into the component envelope glycoproteins E2 and E3 (for some alphaviruses, i.e., SINV, SFV, and VEEV, E3 may remain associated with the virion) (73–76). Mature E2-E1 glycoproteins are transported to the plasma membrane where nucleocapsid associates to trigger budding and egress of mature virions (80–86).

Alphaviruses are internalized principally by clathrin-mediated endocytosis, and membrane fusion occurs in endosomes (48–51) (Figure 2). Upon fusion, alphavirus particles disassemble, releasing genomic RNA into the cytoplasm of infected cells. The viral genome is translated to generate the nonstructural (ns) (P1234) and structural polyproteins (52), which enables negative- and positive-strand RNA synthesis and viral replication (detailed in Figure 2). Nascent virions are formed by budding from the host cell plasma membrane (37, 53–86).

## Alphavirus attachment factors and entry receptors

Over the past several decades, many groups have investigated the steps of alphavirus entry given their implications for cellular, tissue, and host species tropism. A host cell surface moiety that is utilized by the virus for internalization into the cell is classified as an entry receptor. This is distinguished from attachment factors (e.g., heparan sulfate proteoglycans or certain lectins) that concentrate virus at the cell surface but are not sufficient to promote or mediate entry (87). The presence of attachment factors and receptors does not guarantee productive infection, although cells lacking such factors likely are not permissive for viral infections.

Experimentally establishing the role of a surface molecule as a bona fide virus receptor requires fulfilment of four conditions (88): (a) a direct physical interaction occurs between the virus and receptor; (b) the receptor facilitates virus internalization into the cell; (c) blocking of the virus-binding site of the receptor with an anti-receptor antibody, mutagenesis of the virus receptor-binding domain, or soluble receptor decoy molecules can inhibit infection; and (d) the cellular tropism of the virus correlates with receptor expression (88, 89). Cells lacking the receptor should be relatively resistant to infection, and expression of the receptor should promote infection.

## Approaches to viral receptor discovery

**Figure 3:**
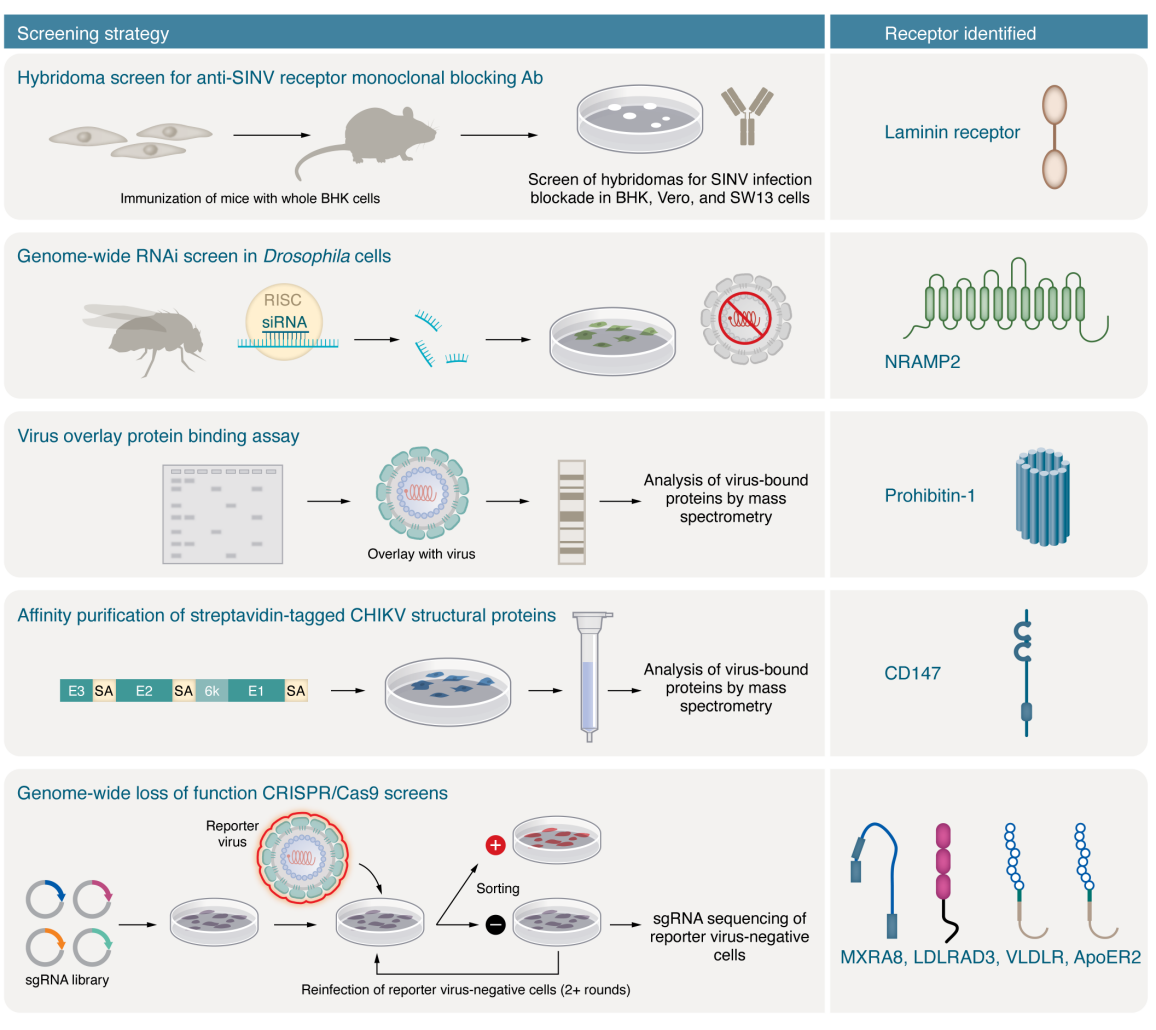
Alphavirus receptor identification and screening methods. Eight alphavirus receptors have been reported to date using the screening methods annotated here and in the main text. Laminin receptor was identified as a receptor for SINV using a blocking mAb screen. NRAMP, a metal ion transporter, was identified as a receptor for SINV in an RNA interference (RNAi) screen in *Drosophila* cells. The mammalian homolog, NRAMP2 (pictured), was shown to mediate SINV binding and infection in mammalian cells. Prohibitin-1 was identified as a possible receptor for CHIKV through virus overlay assays and mass spectrometry. CD147 was identified as a possible receptor for CHIKV by affinity chromatography and mass spectrometry. Four alphavirus receptors were discovered through loss-of-function, negative-selection, CRISPR/Cas9–based genome-wide screens: MXRA8, a receptor for arthritogenic alphaviruses; LDLRAD3, a receptor for VEEV; and VLDLR and ApoER2, which promote cellular entry of SFV, EEEV, and SINV.

Viral receptors have been identified using a range of biochemical, functional, and genetic techniques (Figure 3). Classical methods for host receptor identification included immunoprecipitation and mass spectrometry (90) using antibodies against viral protein epitopes or exogenously placed tags. These methods rely on avid interactions between the viral structural proteins and the cellular receptor target. Blocking of infection with antibodies against host cellular membrane proteins also enabled identification of cell surface viral receptors (90). Perhaps the best-characterized example of this approach is the identification of CD4, a key receptor for HIV-1 infection (91, 92). Antibody blocking experiments also were used to identify ICAM-1 and decay-accelerating factor (DAF) as receptors for human rhinovirus (93, 94) and echoviruses (95), respectively.

Another biochemical technique for identifying virus receptors is the virus overlay protein binding assay (VOPBA). Cell membrane protein fractions from a permissive cell are electrophoresed, followed by blotting onto a nitrocellulose membrane. The blotted membrane is then probed with intact virus (90). Purification of cell membrane fractions and/or mass spectrometry is used to identify putative receptors. Nucleolin was identified as a receptor for respiratory syncytial virus using this method (96). This method is limited by the effect of the denaturing conditions of SDS-PAGE on host proteins and retention of their ability to bind virus particles on the blot.

Genetic screens have transformed the study of virus-host interactions and increased the capacity for identifying receptors. Both gain-of-function (GOF) and loss-of-function (LOF) strategies have been used, although advances in alphavirus receptor identification so far have been achieved using LOF strategies (97–99). One GOF approach used to identify receptors from unrelated viruses is to transduce or transfect a cDNA library derived from a virus-permissive cell line into a non-permissive cell line for the specific virus of interest (90). Cells that ectopically express a virus receptor using this method will be infected, allowing for identification of the viral receptor. The poliovirus receptor was identified using GOF screens with genomic DNA or cDNA libraries from HeLa cells, and expression in mouse cells resistant to poliovirus infection (100). Another method that could be used for GOF screens is with a catalytically inactive Cas9 (dCas9). In this approach, dCas9 is fused to a transcriptional activator, and single-guide RNAs (sgRNAs) target specific gene promoter sequences to induce expression without introducing mutations (101, 102). Approaches that use dCas9 for this purpose are commonly referred to as CRISPR activation (CRISPRa) screens (103).

GOF screens have limitations: (a) Induced expression of the receptor must be sufficient for display on the cell surface without an additional requirement for cell type–specific chaperones, partner subunits, or proteins. (b) The approach relies on using non-permissive cell lines that are restricted at the entry level. Some cells may not sustain infection even in the presence of a receptor because of lack of expression of other host genes required for viral replication. In this case, it might be possible to design a virus binding–based flow cytometry screen to identify attachment factors and/or receptors. (c) Lastly, permissive cells may not survive virus infection, making plasmid recovery and receptor identification challenging.

LOF genetic screens have been used to identify receptors for many viruses, including alphaviruses. With the advent of gene silencing (RNA interference) or insertional mutagenesis in human haploid cells, it became possible to disrupt gene expression on a genome-wide scale in mammalian cell culture (104–106). RNA interference (RNAi) screening identified NRAMP/NRAMP2 as a receptor for SINV in *Drosophila* and mammalian cells (107). Retroviral gene trap and transposon-based insertional mutagenesis in human haploid cells enabled discovery of receptors for Ebola and Lassa fever viruses (108, 109). Haploid cell screens, however, are limited by the few mammalian cell types that have a haploid or near-haploid karyotype, which may not be suitable for some viruses. Commonly used haploid cell lines include the chronic myeloid cell line KBM7 (106) and its derivative, HAP1 (108, 110), and human (111) and mouse embryonic stem cells (112, 113).

More recently, genome-wide CRISPR/Cas9 LOF screens have been performed to identify viral receptors. The editing of gene alleles via introduction of nonsense mutations or deletions by CRISPR/Cas9 often produces a more complete and specific LOF phenotype, which results in a greater signal-to-noise ratio and fewer false positives than other screens (114–117). The ease of designing sgRNAs to specific genes, combined with the incorporation of multiple sgRNAs per gene that span the genome and optimized post-screen (MAGeCK) software analysis (117–120), has enabled the identification of host factors crucial for virus replication, including receptors. A permissive cell line is transduced with a genome-wide library of sgRNAs that target multiple sites in the coding region of each host gene. The transduced cells are inoculated with virus, and cells that lose the ability to support infection are sequenced to identify the edited genes associated with loss of infection. Validation of target genes with multiple sgRNAs that were used in the original CRISPR/Cas9 screen library or designed de novo along with complementation with seed sequence variants has streamlined the process of identifying genes required for infection. This approach was used to identify four new alphavirus receptors: MXRA8 (CHIKV, MAYV, RRV, ONNV, and SFV), LDLRAD3 (VEEV), VLDLR (EEEV, SFV, and SINV), and ApoER2 (EEEV, SFV, and SINV) (97–99). CRISPR/Cas9 screens focused on cell surface proteins (Surface-OME screen) might be an even more efficient tool for identifying viral receptors in the future (99, 121), as this sgRNA library targets genes encoding only surface proteins (1,344) instead of the significantly higher number of genes (18,421) in the genome-wide library (121).

### Types of selection screens and their limitations.

For highly cytolytic alphaviruses, LOF screens enable a straightforward selection of cell survival. The outgrowth of the few virus-resistant cells is stringent, as resistant cells must survive multiple rounds of exposure and infection. Nonetheless, the LOF screening approach has limitations: (a) Surviving gene-edited cells may contain sgRNAs that affect expression of genes associated with post-entry viral processes including translation, replication, and cell death. Thus, extensive validation of the mechanism of action is required. (b) It may be challenging to identify viral receptor genes that are required for cell survival or homeostasis, as loss of expression may be lethal to the cell. (c) Several viruses, including alphaviruses, may bind more than one receptor on a given permissive cell type. Editing of a single receptor gene may not prevent infection and virus-induced cell death. As an alternative approach to cell survival screens, FACS-based selection has been used (97). FACS-based selection enables the isolation of cells with either low or high levels of viral antigen expression, making it possible to identify host factors that, when gene-edited, result in diminished or enhanced infection.

## Alphavirus attachment factors

Before the implementation of genetic screening modalities described above, three types of alphavirus attachment factors had been identified: heparan sulfate, C-type lectins, and phosphatidylserine receptors (Table 1). All three classes serve as attachment factors for both Old and New World alphaviruses in human, other mammalian, and avian cells (122–126). The requirement for attachment factors on a cell is not absolute, as some receptors that are expressed at high levels and bind to specific alphaviruses might preclude a need for separate attachment factors (127, 128). Moreover, the role of alphavirus attachment factors (83–86) in tissue tropism and pathogenesis is not fully characterized.

### Heparan sulfate.

Heparan sulfate (HS), a negatively charged glycosaminoglycan, was first identified as an attachment factor for SINV in baby hamster kidney (BHK) cells (122). This discovery was based on the observation that pretreatment of cells with heparin augmented SINV binding (129). However, this finding was determined to be a cell culture adaptation and associated with a mutation in E2 (G70L) (122). Other alphaviruses (e.g., CHIKV, SFV, and RRV) also can utilize HS as an attachment factor as an adaptation after virus passage. A G82R substitution in domain A of CHIKV E2 augments viral interaction with HS (122) compared with the glycine residue in clinical isolates (123). SFV also bound liposomes containing heparin after passage of the virus in cell culture (124). RRV adaptation to HS was demonstrated in chicken fibroblasts, even though birds are not a reservoir of RRV (125, 126). Charge mutations in E2 domain B residue 218 (N218K and N218R) enhanced RRV infection in avian cells and binding to heparin-Sepharose affinity columns. Infection of these RRV mutants was diminished when they were grown in an HS-deficient CHO cell line (125, 126). Cryo–electron microscopy (cryo-EM) imaging experiments with RRV E2-N218R revealed that HS binds the most distal portion of E2 (130).

Despite the increase in binding to HS following cell culture passage, adapted alphaviruses were attenuated in mice (123, 131–134). One explanation is that viruses that bind to HS become trapped at the cell surface during entry or egress (135). Alternatively, HS binding might target alphaviruses to cells that are non-permissive or less permissive, preventing productive infection and spread (132). One exception to this paradigm is EEEV. Clinical EEEV isolates can use HS as an attachment factor without adaptation (131). Mutagenesis of three conserved lysine residues in domain A of E2 (E2-71, E2-74, and E2-77) abrogated EEEV binding to HS and decreased neurovirulence in mice (131). Neurovirulence conferred by HS binding also has been reported with other neurotropic alphaviruses (136–138). Although the magnitude of viremia is reduced for HS-binding alphaviruses, the effects on pathogenesis are counterbalanced by enhanced infectivity in specific cells of the central nervous system, possibly neurons or glial cells. Thus, HS-binding neurotropic alphaviruses that do not require sustained viremia to access the central nervous system may have a greater potential for virulence. More studies are needed to distinguish how HS adaptations affect alphavirus infection, tropism, and pathogenesis in mammalian and mosquito hosts.

### C-type lectins.

C-type lectins are a family of proteins that have roles in cell migration (139) and glycan pattern recognition (140). Their discovery as alphavirus attachment factors was based on the observation that yeast mannan, a competitive inhibitor of carbohydrate binding to C-type lectins, inhibited SINV infection of macrophages (141). Two WEE complex alphaviruses, SINV and Aura virus, as well as VEEV reportedly use C-type lectins, including DC-specific ICAM-3–grabbing non-integrin (DC-SIGN) and liver-specific ICAM-3–grabbing non-integrin (L-SIGN), as attachment factors (141–143). Cells expressing DC-SIGN or L-SIGN supported increases in SINV binding and infectivity (141). However, this phenotype was observed only with SINV propagated in mosquito cells, which produce high- and pauci-mannose N-linked glycans compared with the complex N-linked glycans of mammalian cells (144). These and other experiments demonstrated that mannose processing on N-linked glycans of the structural proteins affects alphavirus interactions with DC-SIGN or L-SIGN. Thus, high-mannose glycosylation of alphavirus glycoproteins may influence the attachment ligand interactions and tropism early during infection in mammalian hosts (145). Nonetheless, to date, there is no direct correlation between C-type lectin engagement and alphavirus infection in vivo.

### Phosphatidylserine receptors.

Many enveloped viruses incorporate lipids into their bilayers during virion assembly that can bind to host cellular ligands and modulate inflammatory responses. Phosphatidylserine (PtdSer) is one such cell-derived lipid that viruses use to disguise themselves as apoptotic bodies in a process termed apoptotic mimicry (146). Several PtdSer receptors have been identified that enhance virus entry or promote infection through signaling cascades (146). The role of PtdSer receptors in viral attachment was identified by the discovery that detergent-mediated lipid depletion and inactivation of vaccinia virus were rescued by incubation with PtdSer liposomes (147). PtdSer receptors enhance infection of a range of enveloped viruses, including CHIKV, RRV, SINV, and EEEV (146). The T cell immunoglobulin mucin (TIM) domain family proteins (TIM-1, TIM-3, and TIM-4) were the first PtdSer-binding receptors proposed as attachment factors for alphaviruses (127, 148). Infection of pseudotyped vesicular stomatitis viruses displaying alphavirus E1-E2 glycoproteins was increased in cells expressing TIM-1 and decreased in the presence of soluble PtdSer-containing membranes. This suggested infectivity depended on TIM-1 binding of PtdSer as opposed to a direct interaction with the virus glycoproteins (148). Ectopic expression of TIM-1 also increased RRV uptake and infection in cells, and this phenotype was blocked by incubation with an anti–TIM-1 antibody (148). A similar function of TIM-1 was demonstrated in CHIKV. Point mutations in the PtdSer binding site of TIM-1 resulted in reduced cell binding, entry, and infection with CHIKV, and ectopic expression of TIM-1 enhanced CHIKV infection of keratinocytes. (149). A separate study using pseudotyped viruses expressing SINV structural glycoproteins (150) expanded the list of PtdSer-binding proteins potentially used by alphaviruses to include milk fat globule–epidermal growth factor–factor 8 (MFG-E8) and growth arrest–specific gene 6 (Gas6), two soluble adaptor molecules that engage PtdSer (150). Another PtdSer receptor, CD300a, also enhanced the binding of pseudotyped virus displaying SINV E1/E2 to cells (150). Finally, the TAM receptor AXL was shown to enhance SINV and RRV infection in a PtdSer-dependent manner by binding the serum proteins Gas6 and protein S, which in turn bound PtdSer displayed on viral membranes (127, 128). Despite many different PtdSer receptors being implicated in alphavirus attachment and infection, to date, there are no data on their effects on alphavirus infection or tropism in vivo.

## Alphavirus entry receptors

Eight different cell surface proteins have been described as putative entry receptors for alphaviruses in mammalian, avian, arthropod, and nematode species (Table 1), although the level of supporting evidence for each receptor varies. The first two receptors for SINV (laminin receptor and NRAMP2) were identified using a library of blocking monoclonal antibodies (mAbs) and RNAi (107, 151, 152). The third and fourth molecules (prohibitin-1 and CD147) were identified by 2-dimensional virus overlay binding or coimmunoprecipitation assays coupled with mass spectrometry. Four additional receptors for CHIKV (MXRA8) and other arthritogenic alphaviruses VEEV (LDLRAD3), EEEV, SFV, and SINV (VLDLR and ApoER2) were identified more recently using CRISPR/Cas9 screens (97–99). In this section, we review the evidence for these proteins as alphavirus receptors.

### Laminin receptor.

The 67-kDa high-affinity laminin receptor, which functions in both development and tumor metastasis, was the first alphavirus receptor identified based on mAb blocking screens (151). Anti–laminin receptor antibody inhibited SINV binding to BHK, Vero, and SW13 cells, and SINV binding was enhanced in CHO cells overexpressing laminin receptor (151). Despite these data, direct binding between laminin receptor and SINV or evidence for laminin receptor–mediated internalization of SINV has not been documented. Although a more recent study suggests that the carboxyl-terminal domain of laminin receptor interacts with VEEV E2 (152), an effect on infection with this encephalitic alphavirus has not been described. Moreover, there are no data in vivo on the contribution of the high-affinity laminin receptor to SINV infection or pathogenesis.

### NRAMP/NRAMP2.

Natural resistance–associated macrophage protein (NRAMP) was identified as an insect cell receptor for SINV using RNAi screening in *Drosophila* cells, and its mammalian ortholog, NRAMP2, was proposed as a receptor for SINV in mammalian cells (107). Gene silencing of *Drosophila* NRAMP resulted in decreased infection of SINV strains in flies (107). As transfection of SINV RNA directly into *Drosophila* cells bypassed a requirement for NRAMP, this protein was hypothesized to function during alphavirus entry. Direct virus binding to and colocalization with NRAMP were demonstrated by coimmunoprecipitation and confocal microscopy experiments. SINV infection also was reduced in NRAMP2-deficient mouse embryonic fibroblasts. Iron treatment, which downregulates NRAMP/NRAMP2 protein expression, resulted in loss of SINV infection in several insect and mammalian cell lines. In contrast, infection of a chimeric alphavirus displaying the RRV envelope proteins was insensitive to iron treatment or NRAMP2 deletion. The role of NRAMP2 in SINV pathogenesis in mammals remains uncertain, as NRAMP2-knockout mice die shortly after birth and naturally occurring functional mutations cause severe anemia, potentially confounding results (153, 154). Thus, the physiological importance of NRAMP2 for SINV and other alphaviruses remains to be demonstrated.

### Prohibitin-1.

Prohibitin-1 (PHB1) regulates cell proliferation and mitochondrial integrity, and was one of the first proposed CHIKV receptors (155). PHB1 was identified as a candidate binding partner for CHIKV in microglial cells using 2-dimensional virus overlay binding assays and mass spectrometry (156). An interaction between CHIKV E2 protein and PHB1 was confirmed by confocal microscopy and coimmunoprecipitation studies (156), although direct binding of purified, recombinant PHB1 and CHIKV was not demonstrated. Incubation of cells with increasing concentrations of an anti-PHB1 antibody or PHB1 siRNA decreased CHIKV infection of cells (156). Although these data suggest that PHB1 might act as a receptor for CHIKV, its effects on virus binding and internalization in cells have not been reported. Moreover, at present, there are no data in vivo establishing a key role of PHB1 in CHIKV infection.

### CD147.

The CD147 protein complex was identified as a possible entry factor for alphaviruses using an affinity purification scheme with CHIKV virus-like particles (VLPs) and 293T cell lysates and subsequent mass spectrometry (157). Follow-up experiments showed that CRISPR/Cas9–mediated deletion of CD147 or its complex protein partner CLS1A5 resulted in a moderate reduction in CHIKV reporter virus (CHIKV isolate LR-2006 East/Central/South African strain) binding and infection in human 293T cells. Other arthritogenic and encephalitic alphaviruses (e.g., RRV, SINV, WEEV, and EEEV) showed similar loss-of-infection phenotypes following deletion of CD147 with or without CLS1A5, with SINV showing the greatest reductions (157). Unexpectedly, this phenotype was not observed with a West African strain of CHIKV. Moreover, direct binding of CD147 to CHIKV or other alphaviruses was not shown, and correlations between CD147 expression and CHIKV infection were not reported. Clearly, more experiments are needed to establish the role of CD147 in alphavirus cell entry, infection, and pathogenesis and whether it truly acts as a bona fide entry receptor.

### MXRA8.

Matrix remodeling associated 8 (MXRA8; also called DICAM, ASP3, or limitrin) was identified as a receptor for CHIKV and other Semliki Forest complex members (SFV, ONNV, RRV, and MAYV) using a CRISPR/Cas9 screen in mouse 3T3 cells (97). MXRA8 is an adhesion molecule present in mammals, birds, and amphibians and expressed on epithelial, myeloid, and mesenchymal cells (158–161). It was the top hit on a flow cytometry–based CRISPR/Cas9 LOF screen in 3T3 cells (97) and fulfilled the criteria as a receptor: (a) recombinant MXRA8 directly bound with CHIKV with high (~80 nM) affinity; (b) MXRA8 facilitated CHIKV binding and internalization into target cells; (c) CHIKV infection was reduced following blockade with anti-MXRA8 mAbs, knockout of MXRA8 gene expression, or incubation with soluble MXRA8 receptor decoys; and (d) CHIKV infection correlated with cell surface expression of MXRA8 (97).

The role of MXRA8 in pathogenesis of arthritogenic alphavirus infection was demonstrated in mice (97, 162, 163). Coinjection of a MXRA8-Fc decoy receptor with CHIKV-AF15561 or ONNV diminished infection and swelling in the foot and muscle (97). Passive transfer of blocking anti-MXRA8 mAbs reduced CHIKV infection in joint-associated tissues (97). To confirm these results, CRISPR/Cas9–gene-edited mice with mutant or knockout *Mxra8* alleles were generated. MXRA8-deficient primary fibroblasts sustained reduced CHIKV infection in cell culture, and MXRA8-deficient mice showed markedly reduced infection and swelling of musculoskeletal tissues after inoculation with CHIKV, MAYV, RRV, or ONNV (163). These studies establish a key role for MXRA8 in the pathogenesis of multiple alphaviruses and suggest that pharmacological targeting of this protein (e.g., with soluble decoy receptors) might mitigate disease.

The complex between MXRA8 and CHIKV was solved by cryo-EM (164–166). The ectodomain of MXRA8 is composed of two immunoglobulin-like domains oriented in a unique disulfide-linked head-to-head arrangement. MXRA8 binds to residues in E2 and E1 of CHIKV by wedging into a cleft created by two adjacent heterodimers in one trimeric spike and engaging a neighboring spike. Contact residues were identified in both domains 1 and 2 of MXRA8 (164, 165). Mutations in some of the tested MXRA8 contact residues were associated with reduced binding and infection (164, 165). These structural studies provided insight into how MXRA8 binds CHIKV and create a path for developing alphavirus decoy receptors or entry inhibitors.

### LDLRAD3.

Using a CRISPR/Cas9 screen in N2a neuronal cells, low-density lipoprotein receptor class A domain–containing 3 (LDLRAD3) was identified as a receptor for VEEV but not other encephalitic or arthritogenic alphaviruses (98). Before the screen, to reduce background infection, N2a cells were edited to lose expression of *B4galt7*, which encodes an enzyme required for the biosynthesis of HS and other glycosaminoglycans (98). The library of Δ*B4galt7* N2a cells was inoculated with a chimeric SINV-VEEV-GFP that encodes the nonstructural genes of SINV, the structural genes of VEEV, and green fluorescent protein (GFP) (98). This allowed for the screen to be performed at a lower biosafety containment level. In this LOF screen, the few cells lacking GFP expression (or infection) were sorted, propagated, and then reinoculated with SINV-VEEV-GFP. The top hit was LDLRAD3, a conserved plasma membrane protein of the LDL scavenger receptor family found in mammals, birds, reptiles, amphibians, and fish (98).

VEEV infection was abolished in LDLRAD3-knockout cells and restored in cells complemented with LDLRAD3 (98). Overexpression of LDLRAD3 was associated with higher levels of infection, and cells expressing LDLRAD3 had a higher level of VEEV binding and internalization compared with knockout cells (98). Domain 1 (D1) of LDLRAD3 bound directly to VEEV but not to CHIKV VLPs (98), and VEEV infection was blocked by LDLRAD3-D1-Fc decoy molecules (98). Treatment of cells with anti-LDLRAD3 polyclonal antibodies also blocked VEEV infection (98).

The role of LDLRAD3 in VEEV pathogenesis was demonstrated in mice (98). Injection of LDLRAD3-D1-Fc was associated with near-complete survival of mice infected with VEEV compared with the 100% death rate of animals injected with control protein. VEEV infection in serum, spleen, and brain of mice given LDLRAD3-D1-Fc was remarkably (>3 log_10_) lower than that in animals given control protein. Gene deletion of LDLRAD3 in mice was associated with 100% survival after infection with multiple VEEV strains, whereas congenic wild-type mice succumbed rapidly to infection (98).

A cryo-EM structure revealed that D1 of LDLRAD3 binds to domains A and B of E2 and the fusion loop in E1 of VEEV by wedging into a cleft created by two adjacent E2-E1 structural protein heterodimers (167, 168). Atomic modeling of this interface was supported by mutagenesis studies and anti-VEEV antibody binding competition assays (167). Interestingly, VEEV engages LDLRAD3 in a manner that is similar to the way CHIKV binds to MXRA8, but with a much smaller interface (167), even though LDLRAD3 and MXRA8 are not structurally related.

### VLDLR and ApoER2.

Very low-density lipoprotein receptor (VLDLR) and apolipoprotein E receptor 2 (ApoER2), two closely related scavenger family receptors, were identified recently as alphavirus receptors in vertebrate and invertebrate cells (99). VLDLR and ApoER2 are members of the LDL receptor family and mediate endocytosis of lipoproteins and other ligands (169). Both receptors were identified using a CRISPR/Cas9 screen in 293T cells (99). The E2 and E1 glycoproteins of SFV, EEEV, and SINV interacted with the ligand-binding domains (LBDs) of VLDLR and ApoER2 (99). The LBDs are composed of seven tandem type A domain repeats arranged in head-to-tail fashion (169). Ectopic expression of either VLDLR or ApoER2 facilitated cell attachment and internalization of SFV, EEEV, and SINV VLPs (99). Anti-VLDLR antibody and VLDLR LBD–Fc fusion protein inhibited SFV infection of neurons in culture (99). Moreover, administration of a VLDLR LBD–Fc fusion protein prevented fatal SFV infection in neonatal mice (99). The invertebrate receptor orthologs of VLDLR from mosquitoes and worms can serve as entry receptors for SFV and EEEV (99). Nonetheless, infection of CHIKV, VEEV, or WEEV was not supported by VLDLR or ApoER2. The contribution of VLDLR and ApoER2 to alphavirus pathogenesis has yet to be determined, as knockout mice were not evaluated. It remains unclear whether there is cell type– and/or tissue-specific utilization of VLDLR and ApoER2, or whether other members of this protein family also can act as receptors for different alphaviruses.

## Conclusions and research prospects

Cell attachment and internalization are requisite events in the infection cycle of every virus. The identification of surface proteins that serve as attachment factors or receptors advances our understanding of virus biology and could prompt the development of new antiviral therapies that target the entry step(s). These discoveries also enhance our understanding of viral pathogenesis, particularly, the tropism of viruses for specific organs and hosts, and how adaptations might occur.

The advances in CRISPR/Cas9 screening approaches have led to the identification of four new alphavirus receptors (MXRA8, LDLRAD3, VLDLR, and ApoER2) within four years, with two of these receptors (MXRA8 and LDLRAD3) having a clearly defined role in alphavirus tropism and pathogenesis in vivo. The high-resolution structural analysis of virus-receptor complexes (MXRA8-CHIKV, LDLRAD3-VEEV) along with detailed molecular analysis (SFV/EEEV-VLDLR) has enabled the generation of soluble decoy receptors that inhibit infection in cell culture and animals. Using LOF and GOF CRISPR/Cas9–based screens, additional alphavirus receptors may soon be discovered.

Many outstanding questions remain about alphavirus receptors: (a) What is their role in the pathogenesis of alphavirus infection in different hosts? (b) Is there cell type–specific receptor utilization that dictates tissue tropism and disease manifestations? (c) Are there separate viral receptors in endosomes akin to Niemann-Pick C1 protein for Ebola virus (108, 170, 171) that are required for fusion and nucleocapsid penetration into the cytoplasm? (d) What are the entry pathways utilized by this suite of alphavirus receptors? (e) Do genetic polymorphisms in alphavirus receptors affect the susceptibility of humans or other species to alphavirus infections? Answering these fundamental questions will address gaps in our knowledge about the alphavirus entry pathway and may allow for the generation of new classes of countermeasures that target this critical initial step in the alphavirus infection cycle.

## Figures and Tables

**Table 1:**
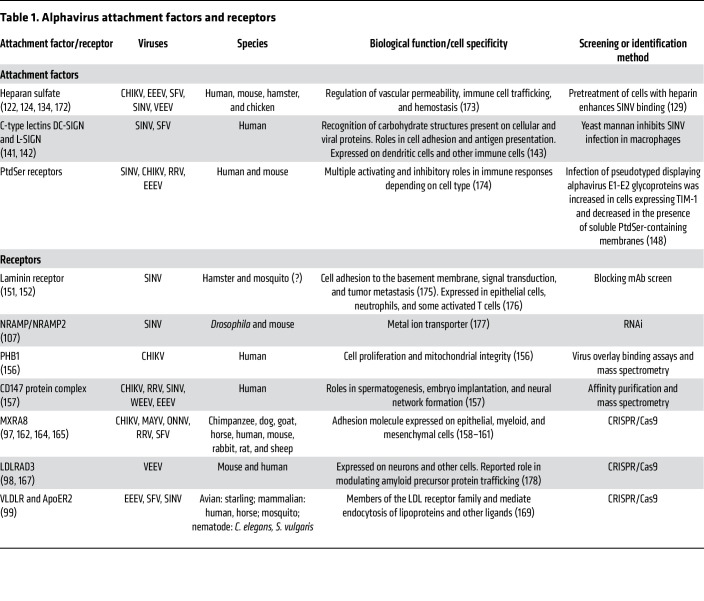
Alphavirus attachment factors and receptors

## Version 1 01/17/2023

Electronic publication
